# Impact of COVID-19 Quarantine on Advanced Hemorrhoidal Disease and the Role of Telemedicine in Patient Management

**DOI:** 10.3390/jcm9113416

**Published:** 2020-10-25

**Authors:** Paola Campennì, Angelo A. Marra, Lorenzo Ferri, Raffaele Orefice, Angelo Parello, Francesco Litta, Veronica De Simone, Marta Goglia, Carlo Ratto

**Affiliations:** 1 Proctology Unit, Fondazione Policlinico Universitario A. Gemelli IRCCS, Largo A. Gemelli 8, 00168 Rome, Italy; campennip@hotmail.it (P.C.); angeloalessandromarr@libero.it (A.A.M.); lorenzoferri.med@gmail.com (L.F.); r.orefice89@gmail.com (R.O.); francescolitta83@yahoo.it (F.L.); veronicadesimone@libero.it (V.D.S.); martagoglia@hotmail.com (M.G.); carloratto@tiscali.it (C.R.); 2 Department of General Surgery, Università Cattolica del Sacro Cuore, Largo Francesco Vito 1, 00168 Rome, Italy

**Keywords:** COVID-19 quarantine, telemedicine, hemorrhoidal disease management, hemorrhoids risk factors, lifestyle habits, sedentary, weight gain, pandemic effects on quality of life

## Abstract

The aims of this population study were to assess the lockdown impact on patients waiting for hemorrhoidal surgery, and the role of telemedicine in patient management. All patients on our waiting list for hemorrhoidal surgery were considered. Eligible patients were contacted by phone. Rørvik score was evaluated and compared to the baseline score. Univariate and multivariate analyses were performed. A numeric rating scale was used to estimate patient satisfaction regarding telemedicine. One-hundred and ten patients were found to be eligible. Finally, 103 patients were included in the study of whom 16 (15.3%) were already considered for priority surgery. Patients waiting for a longer time showed significantly worse scores (*p* < 0.001). After telemedicine evaluation the priority waiting list increased by 43.8% (plus 23 patients). Weight loss of at least 3 kg and physical activity were found to be protective factors (*p* = 0.02 and *p* = 0.002 respectively). A high grade of satisfaction (almost 80%) towards telemedicine was registered. COVID-19-related delays are linked to a deterioration of hemorrhoidal symptoms and patients’ well-being. Weight and lifestyle changes were deemed key factors in determining disease severity. Telemedicine was a valuable tool to evaluate and re-evaluate patients waiting for hemorrhoidal surgery and was considered satisfactory by patients.

## 1. Introduction

The impact of COVID-19 on surgical activities has been devastating [1]. All clinical centers postponed and gradually rescheduled activities, prioritizing procedures based on disease severity and clinical urgency, as suggested by several surgical societies [2]. Proctological surgery has been significantly reduced. In this scenario, telemedicine has been promoted as a possible alternative tool to evaluate or re-evaluate patients, to minimize exposure risks and to establish priorities, ensuring appropriate treatment for all patients [3].

Most proctologic diseases are generally benign and non-urgent, but, if neglected, may worsen or complicate, which seriously affects patients’ quality of life. Hemorrhoidal disease (HD) is the most common proctologic disease, experienced by more than 50% of the population over the age of 50 [4]. Stress, anxiety, sedentary lifestyle, weight gain, unbalanced diet and constipation or diarrhea are possible factors of HD aggravation [5].

During the COVID-19 quarantine, individuals radically altered their eating, sleeping and lifestyle habits [6]. The effects of this difficult period on proctologic patients’ well-being, psychological and physical health, are not completely known.

The aim of this study was to assess both the impact of the COVID-19 lockdown on HD symptoms and consequences on waiting time in our proctology center. Secondary objectives were to analyze the role of telemedicine in clinical management of advanced HD and patient satisfaction towards this form of clinical evaluation.

## 2. Materials and Methods

### 2.1. Study Design and Approval

This was an observational study conducted and promoted by the proctology unit of the Fondazione Policlinico Universitario A. Gemelli IRCCS in Rome, Italy. The study was drawn up in line with STROBE (Strengthening the Reporting of Observational Studies in Epidemiology) [7] and was conducted in accordance with the Declaration of Helsinki and national guidelines on telemedicine [8]. The protocol was approved by our ethics committee (ID 3339).

### 2.2. Study Setting, Inclusion and Exclusion Criteria

All patients affected by advanced HD who were on the waiting list for surgery were considered for this study. At first visit, healthy lifestyle, fiber-rich diet, adequate water intake and, when indicated, flavonoid therapy were generally suggested as a bridge to surgical treatment. Ordinarily, the waiting time for operation ranges from five to eight months. However, our waiting list had a particular section for clinical priority patients with chronic anemia, debilitating pain or acute thrombosis, with the aim to reschedule surgery within 30 days. Considering the usual waiting time for hemorrhoidectomy in our practice, patients were divided into two groups: patients already waitlisted five to eight months before the lockdown and those who were inserted on the list two months before the quarantine. Only those aged 18 or older signing the written informed consent for participation and data collection at the first access to the hospital were included in the study. Exclusion criteria were other concomitant colorectal or proctological diseases, cognitive impairment, immunodeficiency, ongoing radiotherapy or chemotherapy, pregnancy, and portal hypertension.

### 2.3. Data Collection

**Figure 1:**
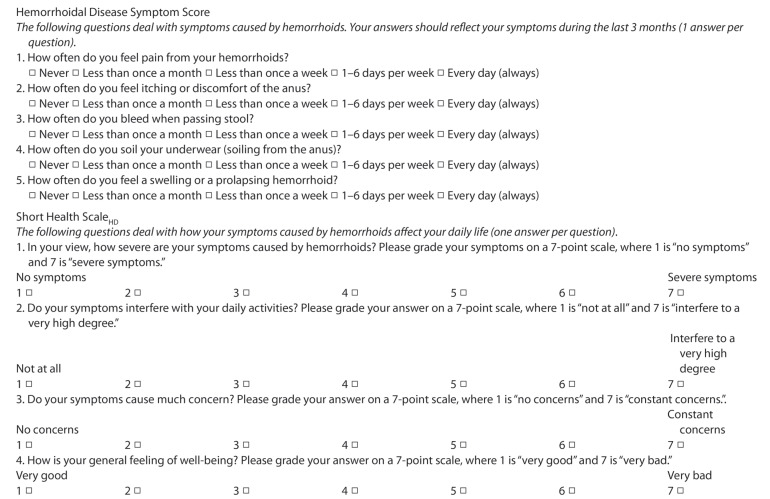
Rørvik score (composed of the Hemorrhoidal Disease Symptom Score, HDSS, and the Short Health Scale-Hemorrhoidal Disease, SHS-HD).

According to common clinical practice in our unit, the hemorrhoidal disease symptom score (HDSS) and short health scale-HD (SHS-HD) by Rørvik et al. [9] were performed on all candidates for hemorrhoid surgery (Figure 1). Questionnaires were translated into Italian and answered, during the visit, by all patients under the supervision of a surgeon to guarantee the complete comprehension of the questions. HDSS were based on five cardinal hemorrhoidal symptoms: they were assessed using patient-reported frequency measure, such as 0 = never; 1 = less than once a month, 2 = less than once a week, 3 = 1–6 days per week, 4 = every day or always (ranging from 0 to 20). Demographic data and disease severity information were retrieved from our computerized filing system.

Between 1 and 7 June 2020, all patients who met inclusion criteria were contacted by phone to evaluate HD symptoms and general health again, using the HDSS and SHS-HD questionnaires, respectively, and answers were compared to those given at the first visit. Other brief questions were asked concerning COVID-19 infection, patient’s weight, physical activity, self-medication and complications or hospitalization due to HD. Additionally, we investigated the use of teleconsultation to manage HD during the COVID-19 emergency, asking about the communication type (phone or video), the frequency of its use and the professional figure contacted (nurse, general practitioner, surgeon, other). Patients were also asked about the grade of satisfaction after telemedicine (using a numerical rating scale: low–moderate–high), and if they would be likely to use it again. The same surgical team performed the first visits and the phone re-evaluation following a shared protocol.

### 2.4. Statistical Analysis

All statistical analyses were performed with SPSS^®^-version 21.0 for Windows^®^ software (SPSS, Chicago, IL, USA). Data are expressed as mean ± standard deviation. Descriptive statistics were used for demographic characteristics of the participants and their behaviors during self-quarantine. Wilcoxon signed-rank test was used to analyze pre- versus post-COVID-19 lockdown continuous data. Continuous and categorical variables were assessed with Mann-Whitney U Test and Chi-square test. Multivariate analysis was performed with binary logistic regression. A *p*-value < 0.05 was considered statistically significant.

## 3. Results

### 3.1. Descriptive Data

From May 2019 to March 2020, 110 patients who visited our proctology center for advanced HD, answered the questionnaires, and were added to the waiting list for surgery. Six patients were unreachable or did not respond to teleconsultations, and one patient was excluded from the study because he was admitted to intensive care for a severe biliary sepsis. Therefore, 103 patients (59 males, 44 females; average age of 51.3 ± 11.9 years) who accepted the invitation to respond the phone interview were included in the present study. Among them, 16 (15.5%) patients were allocated to the priority waiting list, while 87 (84.5%) patients were placed on the ordinary waiting list. All patients were from Central and Southern Italian regions, and no certified COVID-19 infection was reported. Two patients had disabilities (one paraplegic, one blind). No substantial changes in bowel habits were declared. Sixty-seven (65.0%) patients were placed on the ordinary waiting list between May and December 2019, thus, when the lockdown started, they had already finished their ordinary waiting time for surgery.

Patients’ baseline characteristics are detailed in Table 1. Demographic data during COVID-19 quarantine showed that mean patients’ weight increased in 48 (46.6%) patients, was stable in 35 (34.0%) patients and decreased in 20 (19.4%) patients. Fifty-five (53.4%) patients never engaged in physical activity before and after the lockdown, 23 (22.3%) patients declared an increase in sedentary lifestyle habits, 17 (16.5%) patients maintained daily physical activity and 8 (7.8%) patients started this “de novo”. Fifty-eight (56.3%) patients resorted to self-medication for HD symptoms, 5 (4.9%) patients went to the nearest hospital or emergency department, and 3 (2.9%) patients underwent urgent surgery for complicated HD (one case of severe anemia due to acute hemorrhoidal bleeding and two of acute hemorrhoidal thrombosis); two of them were registered on our priority waiting list.

### 3.2. Hemorrhoidal Disease Symptoms

**Figure 2:**
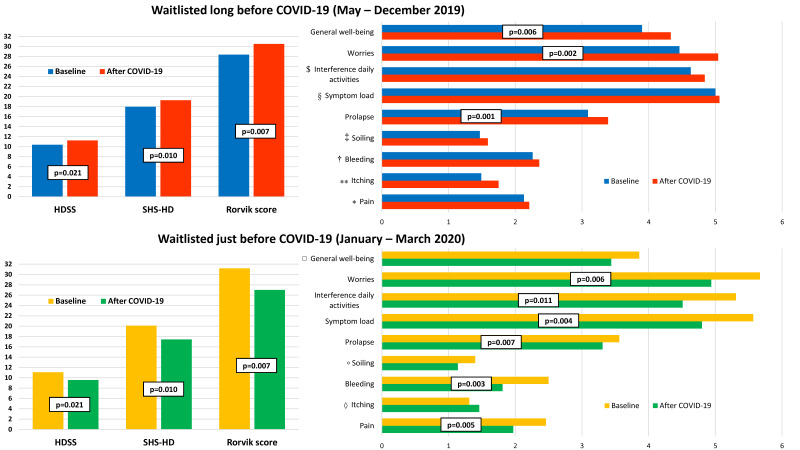
Comparison between Rørvik score in patients waitlisted a long time and just before COVID-19 quarantine. * *p* = 0.461; ** *p* = 0.051; † *p* = 0.298; ‡ *p* = 0.141; § *p* = 0.593; $ *p* = 0.137; ◊ *p* = 0.272; ° *p* = 0.359; □ *p* = 0.172.

At baseline, average Rørvik score was 29.3 ± 8.1 (in detail, mean SHS-HD and HDSS were 18.7 ± 5.6 and 10.6 ± 3.8, respectively). Missing data from patients with disabilities are related to their condition (sensibility or visual impairment). After the lockdown, average Rorvik score, SHS-HD and HDSS showed no significant differences (29.2 ± 9.8, 18.6 ± 6.2 and 10.6 ± 4.6, respectively), excluding a slight worsening in itching (*p* = 0.023). However, scores worsened in patients visited before January 2020 (67 patients), while they improved in the group of patients who visited just before the quarantine (36 patients), as shown in Figure 2 and Table 2. Several items (prolapse, worries, general well-being) showed a significant worsening in patients waitlisted long before COVID-19 lockdown (*p* = 0.001, *p* = 0.002, and *p* = 0.006, respectively). In patients who visited after January 2020, an improvement in some items was registered (pain, bleeding, prolapse, symptoms load, interference in daily activities, worries, respectively *p* = 0.005, *p* = 0.003, *p* = 0.007, *p* = 0.004, *p* = 0.011, and *p* = 0.006).

A weight loss of at least three kilograms and starting physical activity during the COVID-19 lockdown were associated with improved HD scores (Table 2). Another significant relation was evident between a worsening of the HD scores and the use of previous teleconsultation with GP or other surgeons and medical therapy (mainly topical medications). Prolapse aggravated in patients who ceased physical activity (*p* = 0.017, 0.35 ± 0.83 vs. 0.04 ± 0.61). No significant differences between HD scores were reported when considering the hemorrhoid Goligher’s grades and suggested surgical procedures. Other comorbidities and current antithrombotic therapy were without any significant influence.

Univariate analysis showed that longer waiting times and the recourse to teleconsultation were related to a statistically significant poor Rørvik score (*p* < 0.001 and *p* = 0.019, respectively). These correlations were confirmed by the multivariate analysis (*p* < 0.001 and *p* = 0.026, respectively). In the univariate analysis, but not in the multivariate analysis, starting physical activity was associated with an overall improvement (Table 3).

### 3.3. Telemedicine and Changes in the Waiting List

About 80% of the participants referred reported being very satisfied with the telemedicine service and would likely use it again in the future.

Before our telemedicine program, 11 (10.7%) patients had spontaneously used teleconsultation referring to GP or proctology specialist during the COVID-19 quarantine. Voice call, video call or both were used in 7, 3 and 1 cases, respectively. In these patients, the Rørvik score significantly worsened during the COVID-19 quarantine compared to those who did not resort spontaneously to the teleconsultation.

When the lockdown started, 2 patients previously allocated to our priority waiting list underwent urgent surgery, as mentioned before, while 14 were stabilized by appropriate medical therapy. Following our telemedicine program, 11 patients in the whole sample were promptly visited: among them, 9 were transferred from ordinary to priority waiting list and two were confirmed for priority surgery. Therefore, the priority waiting list contained a total of 23 patient names, which signifies an increase of 43.8%.

## 4. Discussion

In our hospital, an entire building, which is still active, has been dedicated to treat COVID-19 patients. In the pre-COVID-19 era the activity in our proctology unit consisted approximately of 80 operations, 140 first visits, 60 anorectal ultrasounds, manometries and high-resolution anoscopy per month. From March 2020 to May 2020 (lockdown period), every elective surgical and outpatient activity at our hospital came to a halt. Consequently, the waiting list was estimated to increase by about six–eight months, considering the months of lockdown and the time needed to reorganize the surgical activity. In the pre-COVID-19 period, our proctologic waiting list was five–eight months and about 60% of patients waited for hemorrhoidal surgery. These data give an idea of the impact of the pandemic on health management and the risk of aggravating or complicating untreated diseases.

Results showed that patients on the waiting list for a longer time had a global deterioration of HD scores, suggesting that a long course of advanced disease is a significant predisposing factor. On the contrary, patients who were included in the list just before the lockdown showed a substantial overall improvement. In this group of patients, the major evidence of improvement was anxiety reduction, probably because they felt reassured by the recent visit and received accurate indications regarding therapies and lifestyle changes. Notably, sedentary lifestyle and overweight are known to be risk factors for HD, due to their close correlation with bowel movements and engorgement of hemorrhoids [5]. A recent US survey of 173 participants reported that lack of sleep, decreased physical activity, snacking after dinner, and eating in response to stress are behaviors linked to weight gain during COVID-19 lockdown [10]. Among our patients, “de novo” starting of physical activity (*p* = 0.002) and weight loss (*p* = 0.020) were factors significantly improving the Rørvik score.

In our study, a worsened Rørvik score was linked to previous voluntary teleconsultation and maintenance therapy. This could be explained by the fact that patients with symptom aggravation look for clinical assistance from their GP or other surgeons and tend to continue already prescribed therapies. Additionally, deterioration of HD might not only be linked to the delay in surgery, but also to relevant lifestyle changes due to the lockdown. Multivariate analysis confirmed that a long course of HD and the previous voluntary recourse to teleconsultation were correlated with a worsening of both HD and well-being. All patients contacted by our team believed that our telemedicine program was a good health instrument and considered it satisfactory. In Italy, after introducing specific telemedicine guidelines in 2012, no public project was initiated which focuses on the spread of telemedicine in the national health system. About eight weeks after lockdown initiation, the ministry of health called for telemedicine-specialized companies to find tools capable of collecting large-scale data to track COVID-19 patients and to ensure the continuity of care for other relevant diseases while minimizing contagion risk. Even if a mobile app has been implemented to anonymously track persons in close contact with COVID-19 patients, no large-scale, non-COVID telemedicine service able to monitor acute and chronic patient’s health exists [11]. This outbreak could provide the right setting to improve integration of telemedicine services in ordinary assistance [12]. Moreover, these services may streamline a re-scheduling of pre-existing operatory lists based on clinical priority. In our unit, updated patients’ conditions, collected through telemedicine and following a prompt visit, led to a substantial modification of our waiting list, which would not have been possible otherwise. Considering all changes after teleconsultation, we aim to treat every patient in our lists in a timely manner.

However, during this study some limits have surfaced. The major point of contention is the impossibility of performing a physical examination, even if partially possible with video assistance. Uncertainty about data collection persists, as even objective data, such as body weight, are self-reported and thus not verifiable. Similarly, variability regarding therapy, especially among patients who resorted to self-medication, should be considered. Of great importance is the methodological gray area regarding the use of telemedicine, stemming from a lack of updated local policies and national guidelines [8]. Another limit of this study was the usage of a questionnaire (Rørvik’s score) that has been validated only in the English language and has been internally translated for clinical practice. As a mitigation, surgeons were present to guarantee adequate comprehension of the questions. Finally, the number of patients enrolled was restricted: this selection bias could be overcome by pooling data in a multicenter setting.

## 5. Conclusions

In conclusion, the COVID-19 lockdown determined a significant worsening of physical and mental health in HD-affected patients waiting for a longer time. Weight gain and physical inactivity are determining factors in score variations. Considering the results, it would be reasonable to expect implications for the choice of surgical strategy before, during and after the intervention, but this requires further studies. Telemedicine is a valid tool to assess patients’ health status and has led to a critical review of our waiting list, which would not have been possible otherwise. Despite its novelty, aided by the contingencies of the pandemic, it has been widely embraced by both patients and surgeons. In consideration of the clinical results, it can be affirmed that telemedicine has a significant impact on the practical management of HD and related complications. As this model of assistance could be applied to other specialties, it would be advisable to implement a long-term plan supported by the national health system to improve implementation of these services.

## Figures and Tables

**Table 1:** Patients’ baseline characteristics.

	*n* Patients (%)
Sex ratio (M:F)	59:44
Age (years)	51.3 (11.9) *
Comorbidities	55 (53.4)
Cardiovascular	35 (34.0)
Endocrine	9 (8.7)
Neurological	6 (5.8)
Gastrointestinal	6 (5.8)
Previous cancer	2 (1.9)
Others	10 (9.7)
Disabilities	2 (1.9)
Blindness	1 (1.0)
Paraplegia	1 (1.0)
Antithrombotic therapy	10 (9.7)
Antiplatelet	9 (8.7)
Anticoagulant	1 (1.0)
Recommended surgical procedure	
Excisional hemorrhoidectomy	48 (46.6)
Dearterialization and mucopexy	25 (24.3)
Dearterialization	2 (1.9)
Mixed procedure †	28 (27.2)

* = values are mean (standard deviation). † Mixed procedure = partial excisional plus non-excisional haemorrhoidectomy.

**Table 2:** Analysis of factors that influenced Rørvik score changes during COVID-19 quarantine.

		Baseline vs. After COVID-19	Delta *	*p*
Time in waiting list before COVID-19	Long time (67) **	28.4(7.8) vs. 30.5(9.9)	2.1(9.0)	<0.001
	Short time (36) †	31.2(8.4) vs. 27.0(9.5)	−4.2(7.8)	
Weight loss	<3 kg (91)	28.9(8.2) vs. 29.6(9.8)	0.8(8.4)	0.020
	>3 kg (12)	33.3(6.2) vs. 26.8(10.6)	−6.5(11.8)	
Starting physical activity	No (95)	29.1(8.1) vs. 29.8(9.7)	0.7(8.7)	0.002
	Yes (8)	32.3(8.5) vs. 22.8(9.8)	−9.5(9.3)	
Voluntary Teleconsultation	No (92)	29.2(8.0) vs. 28.2(9.8)	−1.0(8.6)	0.009
	Yes (11)	30.9(8.9) vs. 38.3(5.5)	7.4(10.0)	

Values are mean (standard deviation). * Delta = Rørvik score evaluated after COVID-19 lockdown minus Rørvik score at baseline. ** Long time = patients waitlisted between May and December 2019; † Short time = patients waitlisted between January and March 2020.

**Table 3:** Univariate and Multivariate analysis of the factors related to Rørvik score variations.

Rørvik Score	Univariate Analysis Odds Ratio (95% CI)	*p*	Multivariate Analysis Odds Ratio (95% CI)	*p*
Long time in waiting list *	0.170 (0.067–0.430)	<0.001	0.135 (0.048–0.375)	<0.001
Weight loss †	0.312 (0.079–1.228)	0.083		
Starting physical activity	0.134 (0.016–1.133)	0.034	0.132 (0.014–1.196)	0.072
Voluntary Teleconsultation	5.598 (1.146–27.350)	0.019	7.412 (1.268–43.338)	0.026
Emergency surgery	2.167 (0.190–24.668)	0.524		
Medication ^§^	2.020 (0.876–4.657)	0.097		
Topical medication	2.148 (0.891–5.177)	0.086		
Antithrombotic therapy	1.670 (0.442–6.310)	0.446		

* Long time in waiting list = patients waitlisted between May and December 2019; † Weight loss = a weight loss of at least 3 kg; ^§^ Medication = oral and topical medications.

## References

[1] COVIDSurg Collaborative (2020). Elective surgery cancellations due to the COVID-19 pandemic: Global predictive modelling to inform surgical recovery plans. Br. J. Surg..

[2] Søreide K., Hallet J., Matthews J.B., Schnitzbauer A.A., Line P.D., Lai P.B.S., Otero J., Callegaro D., Warner S.G., Baxter N.N. (2020). Immediate and long-term impact of the COVID-19 pandemic on delivery of surgical services. Br. J. Surg..

[3] Centers for Disease Control and Prevention Using Telehealth to Expand Access to Essential Health Services during the COVID-19 Pandemic. https://www.cdc.gov/coronavirus/2019-ncov/hcp/telehealth.html.

[4] Gençosmanoğlu R., Sad O., Koç D., Inceoğlu R. (2002). Hemorrhoidectomy: Open or closed technique? A prospective, randomized clinical trial. Dis. Colon. Rectum..

[5] Lohsiriwat V., Ratto C., Parello A., Litta F. (2018). Anatomy, Physiology, and Pathophysiology of Haemorrhoids. Haemorrhoids.

[6] Di Renzo L., Gualtieri P., Pivari F., Soldati L., Attinà A., Cinelli G., Leggeri C., Caparello G., Barrea L., Scerbo F. (2020). Eating habits and lifestyle changes during COVID-19 lockdown: An Italian survey. J. Transl. Med..

[7] Vandenbroucke J.P., von Elm E., Altman D.G., Gøtzsche P.C., Mulrow C.D., Pocock S.J., Poole C., Schlesselman J.J., Egger M. (2007). STROBE Initiative. Strengthening the Reporting of Observational Studies in Epidemiology (STROBE): Explanation and elaboration. Epidemiology.

[8] Italian Ministry of Health (2012). National guidelines on telemedicine. www.salute.gov.it/portale/temi/p2_6.jsp?lingua=italiano&id=2515&area=eHealth&menu=vuoto&tab=2.

[9] Rørvik H.D., Styr K., Ilum L., McKinstry G.L., Dragesund T., Campos A.H., Brandstrup B., Olaison G. (2019). Hemorrhoidal Disease Symptom Score and Short Health ScaleHD: New Tools to Evaluate Symptoms and Health-Related Quality of Life in Hemorrhoidal Disease. Dis. Colon. Rectum..

[10] Zachary Z., Brianna F., Brianna L., Garrett P., Jade W., Alyssa D., Mikayla K. (2020). Self-quarantine and weight gain related risk factors during the COVID-19 pandemic. Obes. Res. Clin. Pract..

[11] Omboni S. (2020). Telemedicine During The COVID-19 in Italy: A Missed Opportunity?. Telemed. e-Health.

[12] Rockwell K.L., Gilroy A.S. (2020). Incorporating telemedicine as part of COVID-19 outbreak response systems. Am. J. Manag. Care.

